# Disturbance of Intracerebral Fluid Clearance and Blood–Brain Barrier in Vascular Cognitive Impairment

**DOI:** 10.3390/ijms20102600

**Published:** 2019-05-27

**Authors:** Masaki Ueno, Yoichi Chiba, Ryuta Murakami, Koichi Matsumoto, Ryuji Fujihara, Naoya Uemura, Ken Yanase, Masaki Kamada

**Affiliations:** 1 Department of Pathology and Host Defense, Faculty of Medicine, Kagawa University, Kagawa 761-0793, Japan; ychiba@med.kagawa-u.ac.jp (Y.C.); ryuta@med.kagawa-u.ac.jp (R.M.); inpathma@med.kagawa-u.ac.jp (K.M.); ryuji@med.kagawa-u.ac.jp (R.F.); uemu@med.kagawa-u.ac.jp (N.U.); yanaken@med.kagawa-u.ac.jp (K.Y.); 2 Department of Neurological Intractable Disease Research, Faculty of Medicine, Kagawa University, Kagawa 761-0793, Japan; kamada93@med.kagawa-u.ac.jp

**Keywords:** BBB, glymphatic system, IPAD pathway, vascular cognitive impairment

## Abstract

The entry of blood-borne macromolecular substances into the brain parenchyma from cerebral vessels is blocked by the blood–brain barrier (BBB) function. Accordingly, increased permeability of the vessels induced by insult noted in patients suffering from vascular dementia likely contributes to the cognitive impairment. On the other hand, blood-borne substances can enter extracellular spaces of the brain via endothelial cells at specific sites without the BBB, and can move to brain parenchyma, such as the hippocampus and periventricular areas, adjacent to specific sites, indicating the contribution of increased permeability of vessels in the specific sites to brain function. It is necessary to consider influx and efflux of interstitial fluid (ISF) and cerebrospinal fluid (CSF) in considering effects of brain transfer of intravascular substances on brain function. Two pathways of ISF and CSF are recently being established. One is the intramural peri-arterial drainage (IPAD) pathway of ISF. The other is the glymphatic system of CSF. Dysfunction of the two pathways could also contribute to brain dysfunction. We review the effects of several kinds of insult on vascular permeability and the failure of fluid clearance on the brain function.

## 1. Introduction

Cerebrovascular insult leads to brain tissue damage followed by cognitive impairment. Patients with vascular cognitive impairment are exposed to several kinds of insult, such as acute cerebral ischemia, hypoperfusion, hypertension or hyperglycemia [1]. Some reports indicate that damage of the blood–brain barrier (BBB) in cerebral vessels plays a role in the pathogenesis of vascular dementia caused by multiple lacunes with endothelial necrosis and leukoaraiosis with perivascular collagen deposition [2,3,4,5]. However, it remains unclear which insult has an effect on the BBB function, or whether there are regional differences in the severity of BBB damage caused by exposure of the insult. In this paper, we review the functions of BBB and blood–cerebrospinal fluid (CSF) barrier (BCSFB) and intracerebral fluid clearance, and the effects of several kinds of insult on vascular permeability.

## 2. BBB and BCSFB

### 2.1. BBB and BCSFB

**Figure 1:**
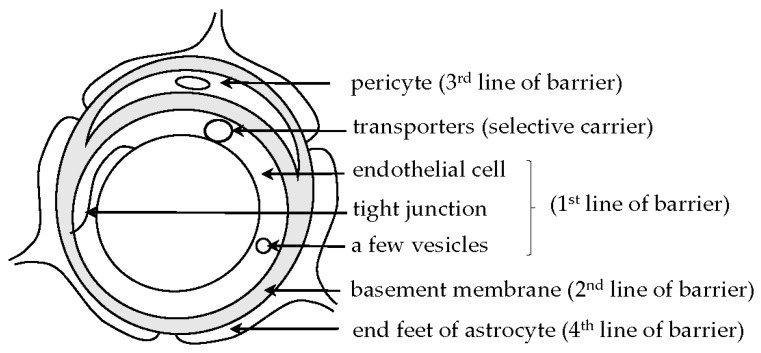
Schematic image of a capillary with the blood–brain barrier is shown. The capillary is composed of endothelial cells with a tight junction and a few vesicles in the cytoplasm (1^st^ line of barrier), basement membrane (2^nd^ line of barrier), a pericyte (3^rd^ line of barrier), and end feet of astrocytes (4^th^ line of barrier), contributing to barrier function of cerebral microvessels. The localization of transporters, contributing to selective carrier function of the vessels, is conceptually shown in the endothelial cell.

It is well known that the entry of intravascular macromolecular substances into the brain is restricted by the BBB present in cerebral vessels (Figure 1), while specific substances including glucose can be transported from the blood into the brain parenchyma through several kinds of transporters in the walls of vessels [6]. The BCSFB is also known and mainly built up by a monolayer of epithelial cells of the choroid plexus separating the blood from CSF [7,8]. However, fenestrated endothelial cells of capillaries in the choroid plexus are permeable to blood-borne macromolecules, allowing the entry of macromolecules from the blood into the brain. Two major barriers, the BBB and BCSFB, may cooperatively play a role in controlling the transport of blood-borne substances into the brain. 

At the BBB, several kinds of endothelial carrier-mediated transport system have been validated, such as: (a) carbohydrate transporters, (b) amino acid transporters, (c) monocarboxylate transporters, (d) hormone transporters, (e) fatty acid transporters, (f) nucleotide transporters, (g) organic anion and cation transporters, (h) other transporters including ones for amines and choline [6]. Most circulating proteins such as albumin, immunoglobulins, and fibrinogen are not transported across the BBB. However, some can transverse the BBB through receptor-mediated transporters. Through endothelial receptor-mediated transporters, transferrin, insulin, and lipoprotein transverse the BBB. The receptor for advanced end products (RAGE) is also known to be located at the luminal membrane of endothelial cells and mediates influx of circulating amyloid-β (Aβ) as well as advanced end products across the BBB. As endothelial efflux transporters, ATP-binding cassette (ABC) transporters such as ABCB1 (also known as P-glycoprotein) and ABCA2 have been validated. They function to prevent brain accumulation of drugs and xenobiotics by active efflux from endothelium to blood. 

**Figure 2:**
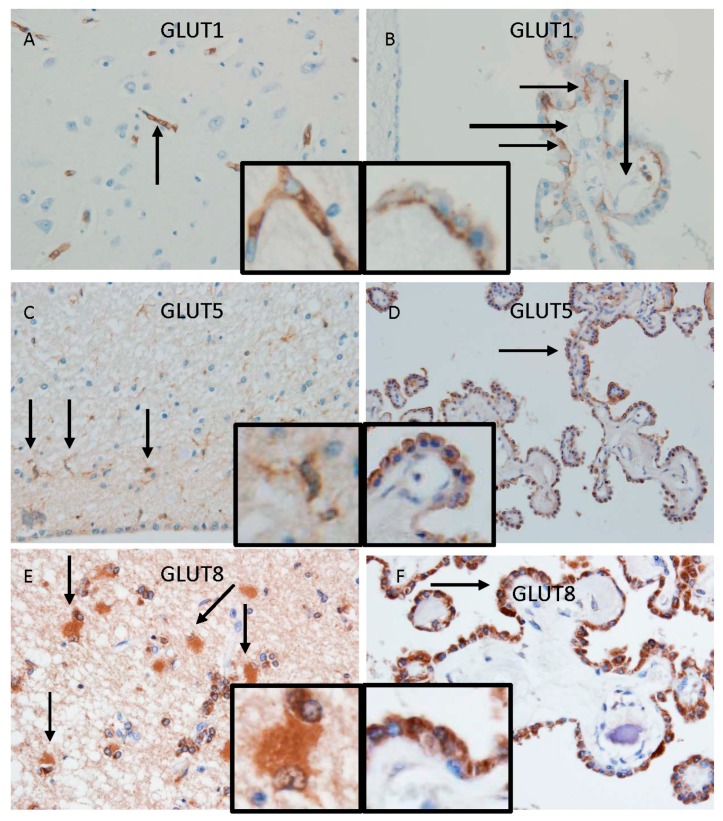
Immunohistochemical localization of representative transporters for glucose (GLUT1) and fructose (GLUT5 and GLUT8) at the BBB and the BCSFB. Microphotographs of immunohistochemical staining using antibodies for GLUT1 (**A**,**B**), GLUT5 (**C**,**D**), and GLUT8 (**E**,**F**) are shown. Immunoreactivity of GLUT1 is observed in the vessel wall in the cortex (**A**: arrow) and in cytoplasmic membrane of epithelial cells (**B**: arrow), but not in the vessel wall (**B**: long thick arrow), in the choroid plexus. Insets; high magnification images show the localization of immunoreactivity in endothelial cells (**A**) and basal cytoplasmic membrane of epithelial cells (**B**). Immunoreactivity of GLUT5 is observed in microglial cells (**C**: arrow) and in cytoplasmic membrane of epithelial cells of the choroid plexus (**D**: arrow). Insets; high magnification images show the localization of immunoreactivity in processes of microglial cells (**C**) and in apical cytoplasmic membrane of epithelial cells in the choroid plexus (**D**). Immunoreactivity of GLUT8 is observed in the cytoplasm of astroglial cells (**E**: arrow) and epithelial cells of the choroid plexus (**F**: arrow). Insets; high magnification images show the localization of immunoreactivity in the cytoplasm of astroglial cells (**E**) and epithelial cells in the choroid plexus (**F**).

BBB and BCSFB are equipped with specific transporters for carbohydrate that contribute to the transport of energy metabolites to the brain parenchyma. Glucose is the most important energy source for the brain. Glucose transporter 1 (GLUT1) transports glucose from either side of the luminal and/or abluminal endothelial membrane according to the concentration gradient (Figure 2A). Accordingly, GLUT1 favors blood-to-brain transport of circulating glucose. Interestingly, GLUT1 is also expressed in epithelial cells of the choroid plexus, while it is not expressed in endothelial cells of the choroid plexus (Figure 2B). Glucose may be transported into the brain through GLUT1 at the BCBFB as well as the BBB. In addition to glucose, fructose can be utilized by brain parenchymal cells as an alternative energy source. Although it remains to be clarified whether intravascular fructose directly affects the brain function, excess fructose intake is thought to be a risk factor for dementia. To clarify whether BBB and BCSFB have the entry route of fructose, the expression of a major representative fructose transporter, GLUT5, and another fructose transporter, GLUT8, in the brain was investigated. Immunohistochemical studies showed the presence of GLUT5 and GLUT8 in the epithelial cells of the choroid plexus, but not in endothelial cells, in brains of humans (Figure 2C–F) and mice [9,10]. In addition, immunoreactivity for GLUT5 is also localized in microglial cells (Figure 2C), while immunoreactivity for GLUT8 is localized in the cytoplasm of astrocytes and microglia around the lateral ventricle (Figure 2E) [11]. These findings support the hypothesis of the transport of intravascular fructose into the brain through the BCSFB, but not the BBB, and suggest some roles of fructose in the brain, such as the source of energy for glial cells under certain circumstances.

With respect to clearance of Aβ, several kinds of transporters or receptors such as low-density-lipoprotein receptor (LDLR), LDLR-related protein 1 (LRP1), LRP2, formylpeptide receptor-like-1 (FPRL1), ABC transporter–A1 (ABCA1), ABCB1, ABCC1, ABCG4, CD36, insulin-degrading enzyme (IDE), and RAGE have been supposed. However, it is unclear whether Aβ are transported transendothelially (BBB) or transepithelially (BCSFB) via these transporters or receptors [6,7,8,12]. An immunohistochemical study using autopsied human brains [13] suggest that immunoreactivity of LDLR, LRP1, LRP2, FPRL1, ABCA1, ABCC1, and ABCG4 was located in the choroid plexus epithelium (BCSFB). Another study reported the immunoreactivity of ABCB1 in the choroid plexus epithelium [14]. Immunoreactivity for LDLR, ABCB1, ABCG2, and RAGE was observed at the BBB. These transporters/receptors expressed at the BBB and BCSFB are supposed to complementarily or cooperatively contribute to the clearance of Aβ peptides from the brain.

### 2.2. Circumventricular Organs (CVOs)

Although the BBB at cerebral vessels restricts the entry of blood-borne macromolecular substances into the brain, the BBB function is defective or absent in certain specific brain regions, known as the circumventricular organs (CVOs) [6]. The question of whether a defective BBB function in CVOs has an effect on areas surrounding these organs has attracted little attention. However, some experimental findings indicate that blood-borne macromolecules can be transferred through vessels with increased permeability in the subfornical organs or choroid plexus, known as non-BBB areas, and move to some areas with an intact BBB function, such as medial portions of the hippocampus and the amygdala, corpus callosum, and periventricular areas [7,8,15]. These experimental findings suggest that blood-borne substances can be transferred in the cerebral parenchyma in close proximity to the CVOs or choroid plexus.

## 3. Intramural Peri-Arterial Drainage (IPAD) Pathway and Glymphatic System

**Figure 3:**
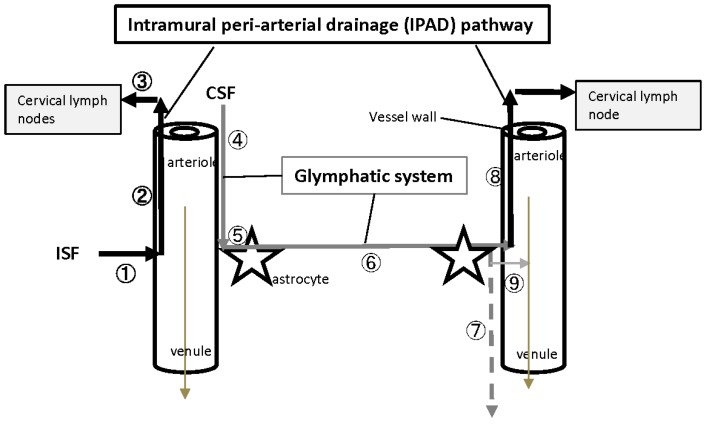
Intramural peri-arterial drainage (IPAD) pathway and glymphatic system in the brain. Clearance of ISF and CSF to the outside of the brain involves both the perivascular IPAD pathway (1,2,3), whereby ISF drains against the blood flow (thinnest brown line in the center of tubes), and paravascular glymphatic system (4,5,6,7), whereby CSF flows in the same direction as blood. Through the IPAD pathway (indicated by thicker solid black lines), ISF (1) flows through the basement membrane of capillary walls, tunica media of arteries, and vessel walls of the internal carotid artery (2), and then drains into the cervical lymph nodes (3). Through the glymphatic system (indicated by thinner gray lines), CSF flows through the para-arterial routes (4), enters interstitial spaces (6) by AQP4-dependent transport through the astroglial cytoplasm (5), flows into the paravenous route (indicated by thinner gray dotted line) (7) or into the perivascular IPAD pathway (8 or 2), and may be drained into the subarachnoid CSF or enter the bloodstream across vessel walls (indicated by thinnest gray line) (9). It is controversial whether or how much the paravenous route (indicated by thinner gray dotted line) (7) is involved in the efflux of toxic intracerebral substances. A critique of the glymphatic system involves the suggested role of AQP4. AQP4 is enriched at CSF interfaces of the brain with expression in astrocytes at the pial surface-facing glia limitans and within perivascular endfeet. It is unclear how perivascular astrocytic AQP4 water channels facilitate a flow of ISF and macromolecules through the brain parenchyma.

Recent studies indicated that both peri- and paravascular clearance occurs in the IPAD pathway [16,17,18,19,20,21,22,23,24] and glymphatic system [25,26,27,28,29,30,31] (Figure 3). Through the IPAD pathway [16,17,18,19,20,21,22,23,24], ISF flows into the basement membrane (BM) of capillary walls and the BM in the tunica media of the arteries and internal carotid artery, and then drains into the cervical lymph nodes. This pathway may be affected by cellular uptake or degradation. On the other hand, through the glymphatic system [25,26,27,28,29,30,31], CSF flows through the para-arterial routes, enters interstitial spaces after aquaporin 4 (AQP4)-dependent transport through the astroglial cytoplasm, drains into paravenous routes, and then may enter the subarachnoid CSF or bloodstream across cerebral vessels. The IPAD pathway and glymphatic system may cooperatively play a significant role in the clearance of intracerebral fluids or toxic substances including Aβ [30,32,33,34,35].

As the hypothesized pathways of fluids may be critical for the maintenance of normal brain functioning, it is reasonable to suggest that an obstacle to the passage of fluids through these pathways may induce several kinds of brain dysfunction, such as amyloid angiopathy or Alzheimer’s disease (AD) [36,37,38]. Aβ peptides in cerebral parenchyma are considered to be eliminated via several kinds of mechanisms: (1) degradation by peptidases or cellular uptake: (2) flow out of the brain parenchyma into the blood through efflux transporters at the BBB and BCSFB: (3) ISF bulk flow clearance including the IPAD pathway and glymphatic system through peri- and paravascular spaces to the cervical lymph nodes. In addition, it is likely that CSF absorption clearance through arachnoid villi and meningeal lymphatic vessels [39,40] and CSF efflux through subarachnoid spaces of olfactory nerves could contribute to Aβ elimination [18,19]. Given that the BBB is functionally and structurally deteriorated with vascular remodeling, drainage through these peri- and paravascular pathways as well as efflux of Aβ by transporters at the vessel wall may be affected. Such vascular dysfunction could induce the deposition of toxic substances including Aβ.

On the other hand, the mechanism of tau clearance remains unclear. Transporters of tau at the BBB have yet to be identified. It is considered that tau is cleared from the brain by degradation, flow of ISF and CSF, and through the glymphatic system [27,41,42]. Also, increased levels of intracellular tau or the aggregation of tau may trigger the release of tau into extracellular spaces, leading to elevated CSF tau levels [30]. The glymphatic system function was reduced by approximately 60% in experimental animals after being subjected to traumatic brain injury, followed by the development of neurofibrillary pathology and neurodegeneration. These findings suggest the contribution of the glymphatic system to tau pathology. Some studies reported the effects of glymphatic system failure on the brain function. Dysfunction of the glymphatic system and subsequent impairment of metabolite circulation aggravates the onset and development of AD [32,33,42]. Diabetes triggers BBB dysfunction and ultimately aggravates cognitive decline through metabolite imbalance due to dysfunction of the glymphatic system [34]. These findings indicate the contribution of glymphatic system failure to the deposition of toxic substances including Aβ and tau, possibly followed by cognitive impairment.

## 4. BBB Damage in Human Brains and Experimental Animal Models for Vascular Cognitive Impairment

The BBB damage has been reported to be present in several kinds of cerebral diseases and play a role in the pathogenesis of vascular cognitive impairment [2,3,4,5,6]. Some factors, such as: (1) aging with or without cognitive dysfunction, (2) hypertension, (3) hyperglycemia, (4) acute ischemia followed by reperfusion, (5) chronic hypoperfusion, and (6) hydrocephalus, are frequently seen in patients suffering from vascular cognitive impairment. In this section, effects of these factors on the BBB function are reviewed.

### 4.1. BBB Changes in Aging With or Without Cognitive Dysfunction

A large-scale meta-analysis study revealed that cerebrovascular permeability increases with age and is further increased in patients showing dementia or white matter lesion [43]. Another study revealed that BBB breakdown is an early finding in aged brains, especially in the hippocampus, and the damage may contribute to the progression of cognitive impairment [44]. BBB permeability was also increased with age in mice [45]. This increase in cerebrovascular permeability was accelerated especially in the olfactory bulb and the hippocampus of 13-month-old senescence-accelerated mouse prone 8 (SAMP8) showing cognitive impairment [7,8,45]. These findings suggest that BBB permeability changes with age, especially in the presence of cognitive impairment.

### 4.2. BBB Changes in Hypertension

It has been reported in human brains that high blood pressure precedes the formation of white matter lesions, possibly accompanied by BBB impairment [46], and that hypertension as well as atherosclerosis and cerebral amyloid angiopathy are the most common causes of BBB lesions [47]. In experiments using animal models of hypertension, BBB permeability to blood-borne horseradish peroxidase (HRP) was increased in the hippocampus of 3-month-old spontaneously hypertensive rats (SHR) and stroke-prone SHR (SHRSP), while no leakage was seen in Wistar Kyoto (WKY) rats without hypertension [48]. Collagen was deposited in the BM of vessel walls showing increased BBB permeability in the hippocampus of SHRSP. It is known that white matter lesions occur in 5-month-old SHRSP rats and that neuronal cell loss occurs with reduced gray matter volume in the hippocampus of SHR at the age of 6 months or older, indicating that SHR may be an appropriate animal model of vascular dementia [49]. These findings suggest that hypertension induces BBB damage with perivascular collagen deposition in the hippocampus and white matter lesions, possibly followed by cognitive impairment.

### 4.3. BBB Changes in Hyperglycemia 

BBB permeability was increased in patients with type 2 diabetes based on gadolinium-enhanced magnetic resonance imaging (MRI) [50]. BBB permeability to sugar derivative tracers such as sucrose was also increased in experimental animals showing hyperglycemia [51]. On the other hand, BBB permeability to blood-borne naïve HRP was not increased in diabetic db/db mice. However, damage to the endothelial glycocalyx was observed in a hyperglycemic state, and moreover, expression of 8-hydroxy-2’-deoxyguanosine (8-OHdG), a marker of oxidative DNA damage, was increased in vessels of diabetic db/db mice compared with controls [52]. Recently, BBB permeability to albumin, which is considered to be glycated in the circulating blood of diabetic mice, was reported to be increased in periventricular areas of mice showing hyperglycemia [53]. These findings suggest that hyperglycemia induces changes in the endothelial glycocalyx and causes oxidative DNA damage of cerebral vessels and, as a result, induces increased permeability to sugar-related tracers such as sucrose and glycated proteins.

### 4.4. BBB Changes in Acute Ischemia Followed by Reperfusion

Using gadolinium-enhanced MRI, increased BBB permeability was observed in acute ischemic stroke cases [54]. Ischemia-modified albumin, which is formed by the production of reactive oxygen species and enters in the brain through an impaired BBB, is likely a useful serum marker for the early diagnosis of stroke [55], suggesting that BBB permeability is increased even in the early stages of acute stroke. BBB permeability to blood-borne HRP was also increased in the hippocampus of a Mongolian gerbil, an experimental model of acute ischemia followed by reperfusion [56]. These findings show that acute ischemia followed by reperfusion enhances BBB permeability in the hippocampus at an early stage.

### 4.5. BBB Changes in Chronic Hypoperfusion

BBB permeability was known to increase in the white matter lesions of Binswanger’s disease patients. BBB damage in white matter lesions due to chronic hypoperfusion, was reported in cerebrovascular and AD patients [57]. BBB permeability to HRP injected intravenously was increased in the corpus callosum of experimental animals showing chronic cerebral hypoperfusion [58]. In addition, collagen was deposited in the thickened BM of the animals. These findings show that chronic hypoperfusion enhances BBB permeability with perivascular collagen deposition in the corpus callosum followed by white matter lesion formation.

### 4.6. BBB Changes in Hydrocephalus

Vascular permeability of blood-borne HRP was examined in an animal model of hydrocephalus, neural cell-specific hypoxia-inducible factor-1α-deficient mice, which showed hydrocephalus with neural cell loss, but it showed no change compared with that of controls [59]. These findings suggest that BBB changes were not confirmed in a state with hydrocephalus. 

From the above results, hypertension and acute ischemia followed by reperfusion enhance BBB permeability in the hippocampus, while chronic hypoperfusion enhances BBB permeability in the white matter. White matter lesions are also induced by hypertension. Hyperglycemia induces changes in endothelial glycocalyx and DNA damage in cerebral vessels, possibly followed by increased BBB permeability to sugar-related tracers. In addition, aging in patients or experimental animals with cognitive dysfunction may contribute to increased permeability in the hippocampus. These results are consistent with the recent paper reporting the presence of BBB breakdown in the hippocampus of aging human brains [44]. However, it remains unclear why the hippocampus is susceptible to hypertension, acute ischemia followed by reperfusion, or aging. 

In addition, genetic factors as well as environmental factors for vascular dementia have been examined. Polymorphisms of Apolipoprotein E, N10-methylenetratahydrofolate reductase, angiotensin converting enzyme, and claudin were reported to be as potential candidates of the genetic factors for vascular dementia although it is unclear whether their polymorphisms are really associated with cognitive impairment [60]. Immunohistochemical studies indicated that expression of claudin family proteins and occludin was altered in AD and vascular dementia brains [61,62]. 

## 5. Target Genes for Vascular Cognitive Impairment

Recent studies revealed that several genes may play an important role in development of vascular cognitive impairment. In this section, it is reviewed how the target genes for vascular cognitive impairment are investigated and identified.

Hypertension is one of the main risk factors for the onset and/or exacerbation of vascular cognitive impairment. BBB damage and neuronal loss with reduced gray matter volume occur in the hippocampus of SHRSP [49]. The hippocampus in the hypertensive rats was reported to be suitable as research object for vascular dementia [49]. In addition, increased vascular permeability in the hippocampus of SHR and SHRSP was seen very limited in the vessel located along the hippocampal fissure of the rats [48]. Accordingly, the vessels located along the hippocampal fissure in the hippocampus of SHRSP were considered to be suitable for investigation of the expression changes of molecules which may have some roles in the pathogenesis of vascular cognitive impairment. 

Microvessels situated along with the hippocampal fissure were microdissected in SHRSP and WKY rats, as a control. RNA was extracted and a microarray analysis was performed to examine the differences in mRNA expression between SHRSP and WKY rats. The assay revealed that the ratio of osteopontin gene expression of SHRSP to that of WKY rats was highest among all genes examined [63]. The mRNA expression of insulin-like growth factor binding protein, transforming growth factor beta receptor II, and Aβ precursor protein of SHRSP was increased in comparison with that of WKY. On the other hand, the estrogen expression of SHRSP was decreased in comparison with that of WKY. 

To confirm the expression changes found in microarray analysis, proteins and mRNA were extracted from the frontal cortex and the hippocampus of SHRSP and WKY rats. Real-time quantitative reverse transcriptase-polymerase chain reaction analysis revealed that the gene expression of osteopontin, matrix metalloproteinase-13 (MMP-13), and CD36 was significantly increased in the hippocampal samples of SHRSP compared with that in the hippocampal samples of WKY rats [7,8,63,64]. The expression levels of MMP-2, MMP-3, MMP-9, AQP1, AQP4, LRP1, occludin, claudin-5, and RAGE were not changed in the hippocampus of SHRSP compared with those of controls. Although the role of increased expression of osteopontin, MMP-13, and CD36 remains to be clarified, the increased expression of them may be an indicator of vascular impairment induced by persistent hypertension in the microvessels of the brain.

## 6. Conclusions

ISF flow has an effect on the passage of blood-borne substances in the brain parenchyma through the CVOs and choroid plexus. Blood-borne macromolecules can be transferred in medial portions of the hippocampus and the amygdala, corpus callosum, and periventricular areas. Two pathways of ISF and CSF are being established. One is the IPAD pathway, whereby ISF drains against the blood flow into cervical lymph nodes through the basal membrane of cerebral arteries. The other is the glymphatic system, whereby CSF flows in the same direction as blood through para-arterial spaces, astroglial cytoplasm with AQP4, interstitial spaces, and paravenous spaces. Dysfunction of the two clearance pathways of ISF and CSF or further increased permeability of vessels without the BBB function in the CVOs and choroid plexus could induce efflux failure of toxic interstitial substances or increased influx of toxic intravascular substances, followed by brain dysfunction including cognitive impairment. These results suggest that improving efflux of intracerebral fluids including the IPAD pathway and glymphatic system could be a potential therapeutic strategy for the prevention of cognitive impairment. In addition, hypertension, acute ischemia followed by reperfusion, chronic hypoperfusion, hyperglycemia, and aging likely contribute to the onset or development of cognitive impairment through BBB dysfunction. Increased expression of osteopontin, MMP-13, and CD36 may be an indicator of vascular impairment induced by persistent hypertension. The development of new therapies is expected against the disturbance of cerebral vessels and the two perivascular and paravascular clearance pathways. It is becoming increasingly important to maintain cerebral vessels in a healthy state for the prevention of cognitive impairment.

## Abbreviation

Aβ	amyloid-β
ABC	ATP-binding cassette
AD	Alzheimer’s disease
AQP4	aquaporin 4
BBB	blood–brain barrier
BCSFB	blood-cerebrospinal fluid barrier
BM	basement membrane
CSF	cerebrospinal fluid
CVO	circumventricular organ
FPRL1	formylpeptide receptor-like-1
GLUT	glucose transporter
HRP	horseradish peroxidase
IDE	insulin-degrading enzyme
IPAD	intramural peri-arterial drainage
ISF	interstitial fluid
LDLR	low-density-lipoprotein receptor
LRP	LDLR-related protein
MMP	matrix metalloproteinase
MRI	magnetic resonance imaging
8-OHdG	8-hydroxy-2’-deoxyguanosine
RAGE	receptor for advanced end products
SAMP	senescence-accelerated mouse prone
SHR	spontaneously hypertensive rat
SHRSP	SHR stroke-prone
WKY	Wistar Kyoto
